# Can morphometric analysis of the fallopian tube fimbria predict the presence of uterine papillary serous carcinoma (UPSC)?

**DOI:** 10.1371/journal.pone.0211329

**Published:** 2019-02-28

**Authors:** Amnon Amit, Edmond Sabo, Avielle Movsas, Yamit Efrat–Tamam, Ari Reiss, Emad Matanes, Geula Klorin

**Affiliations:** 1 Department of Obstetrics and Gynecology, Rambam Health Care Campus, Haifa, Israel; 2 Technion–Israel Institute of Technology, Faculty of Medicine, Haifa, Israel; 3 Institute of Pathology, Carmel Medical Center, Haifa, Israel; Zhejiang University School of Medicine, CHINA

## Abstract

Uterine serous papillary carcinoma (UPSC) is an aggressive tumor, often diagnosed as a metastatic disease and characterized by a high recurrence rate and poor prognosis. UPSC represents a distinct subtype of endometrial cancer which is different in clinical and pathological behaviors from endometrioid endometrial carcinoma (EEC) and resembles more to serous ovarian carcinoma. Since tumors of serous papillary of the ovary are hypothesized to stem from cells of the fallopian tube's fimbria, we hypothesized that UPSC may also origin in the fallopian tubes. In our previous study, using a novel method of computerized morphometry of the fimbrial epithelium we have found significant differences between fimbriae of healthy women and serous ovarian cancer patients. In this study we showed the presence of morphologic differences between twenty-four fimbriae from healthy women, and twenty six fimbriae from uterus cancer (13 from UPSC patients and 13 from EEC patients). All fimbriae reported by the pathologist as "normal" were subjected to a computerized histomorphometric analysis. Two-step method of computerized histomorphometry, i.e. Fast Fourier transformation (FFT) followed by a co-occurrence matrix analysis and an additional analysis of the nuclear symmetry of the tubal fimbrial epithelium were applied. Using these novel methods, we were able to show differences in the morphometric characteristics of the fimbriae in UPSC patients compared to EEC and healthy patients. It is yet to be determined the clinical significance of this observation.

## Introduction

Endometrial cancer is the most common gynecology malignancy in the United States with 47,130 new cases and about 8010 deaths annually [1]. Endometrial tumors are categorized by histological appearance, epidemiology and clinical course [2]. There are two major types of uterine carcinomas. Endometrioid endometrial carcinoma (EEC) is usually of a low to moderately differentiated grade, comprising about 80% of malignancies in uterine cancer cases. It is more prevalent in young patients and correlates with obesity, hyperlipidemia and hyperesterogenism. The second category, including the uterine papillary serous carcinoma (UPSC) and Clear Cell Carcinoma, which are of a high (poorly differentiated) grade and comprise 10–20% of the uterine cancers. The pathophysiology of the classic type of endometrial cancer is well known and is associated with unopposed estrogen. However, the pathophysiology of UPSC has yet to be fully understood and may not be related to hormonal mechanisms [3]. The UPSC variant can be regarded as the uterine counterpart of the ovarian serous carcinoma.

Although UPSC represents less than 10% of endometrial cancers, it causes more than 50% of tumor relapse and death [2]. The 5 years survival of UPSC patients is 18–27%, compared to 85% in the EEC group. In contrast, the majority of the EEC group of patients is diagnosed at an early clinical stage [4] and thus has a better outcome.

The International Federation of Gynecology and Obstetrics (FIGO) stage is used for uterine cancer patients [5–6].

UPSC has a different clinical behavior and molecular profile than EEC. EEC is associated with an inactivation of the tumor suppressor gene PTEN, with mutations in the beta-catenin and KRAS genes and with DNA mismatch repair deficiency. The UPSC cases display a high incidence of P53 mutation and/or overexpression of EGFR and/or HER_2_NEU [2, 7, 8, 9]. UPSC tumors do not express estrogen or progesterone receptors, as compared to EEC tumors [2, 10].

Recent studies in ovarian cancer patients suggested an extra-ovarian origin, possibly from the fallopian tube epithelium that may be a precursor of ovarian serous papillary carcinoma [11,12]. These observations led us to examine the tubal epithelium of patients with ovarian cancer using a novel histomorphometric method which combined for the first time an FFT analysis followed by a gray level co-occurrence matrix analysis [13,14]. By this method we were the first to demonstrate that normal appearing fimbria in patients with serous papillary cancer of the ovary had significant morphological differences (e.g. orientation and texture) from those of the fimbriae of healthy women that were diagnosed as "normal" by the pathologist [13].

In the present study we improved our technique using a second novel method aimed to quantify the loss of nuclear symmetry in the tubal fimbrial epithelium, in patients with uterine serous carcinomas or endometrial cancer, versus healthy women. This method has been already shown to be effective in distinguishing pre-malignant versus malignant conditions in various tissues. [14, 15, 16]

Since the origin of ovarian serous papillary carcinoma was linked to a fallopian tube origin, when considering the morphological and biological similarity between serous papillary carcinomas of ovary and of the uterus, we hypothesized that our morphometric methods will be able to detect interesting differences or similarities between fallopian tubes from healthy women, as compared to fallopian tubes from UPSC and EEC, even though no such association has been reported so far.

## Materials and methods

### Study design

The local Research Ethics Institutional Review Committee of Rambam Medical Center approved the study protocol (0384–13 RMB). The need for patient consent was waived by the ethics committee due to the retrospective nature of the study.

### Study groups

Fifty patients were included in this study: 13 patients with USPC, 13 patients with EEC and 24 healthy women who underwent hysterectomy and salpingectomy (the healthy women group was also included in our previous study [13]). The histological samples from the fallopian tube fimbriae that were diagnosed as "normal" by the pathologist, were submitted to the morphometric analysis, focusing on the fimbria epithelium. The results were used to develop a mathematical classifier for predicting UPSC and EEC in these patients.

#### Image acquisition

Hematoxylin-Eosin -stained histological sections scanned (X600 magnification) and digital images of the fimbrial epithelium obtained using a high-resolution digital camera (Qimaging) attached to a BX51 Olympus microscope. An average of 10 representative microscopic fields of the fimbrial epithelial cells was selected by two observers, blind to the study group category.

#### Image analysis

The Image Pro Plus version 7.0 software was used to select strips of fimbrial epithelium without the underlying stroma. Only strips that contained unfolded, artifact-free epithelium were selected for the analysis. Each image of an epithelial strip was converted to a gray scale and subsequently submitted to a Fast Fourier transformation (FFT) thus creating a two-dimensional plot of frequencies of the pixel bitmaps. These two-dimensional frequency plots reflect changes in the orientation of textures in the tubal epithelial cells. The centers of the 2D frequency plot further transformed into a co-occurrence probability matrix in which the raw pixel matrix of the image is transformed into a probability gray level matrix of frequencies of pairs of neighbor pixels. Four texture variables have been extracted from this matrix. These variables included: homogeneity, correlation, contrast and entropy [13].

The second morphometric method that we have used, include the quantification of low of nuclear symmetries of the fallopian tube epithelial cells. This method included an automatic split of the nuclei by their longest axis passing through the digital center of gravity. Subsequently, each half of the obtained nucleus was measured for a variety of size, shape and textural variables. Then, a ratio of symmetry was calculated for each variable by dividing the smaller value to the larger value of the two halves of the nucleus. Lower indices will be obtained from less-symmetric nuclei [14].

### Statistical analysis

The Kolmogorov Smirnov test was used to evaluate the normality of the groups. The unpaired student T test was applied to compare between each two groups. The p values were then adjusted for the multiple hypotheses problem. A multivariate logistic regression analysis was applied in a stepwise forward mode in order to single out independent morphometric variables that were significantly associated with the diagnostic groups. Using the coefficients of regression obtained from the multivariate analysis and the independent variable values, discriminant scores (DS) were calculated. Best cutoff point in the DS for differentiating between the groups, were found using a Receiver Operating Curve (ROC) analysis. Two-tailed p values ≤ 0.05 will consider to be statistically significant. A leave one out method was also used for cross-validation of the multivariate model. The statistical analysis was performed using the Statistical Package for the Social Sciences version 12 (SPSS, Inc., Chicago, Illinois, U.S.A.).

## Results

### A. Analysis of the morphometric textural features of the fimbriae between study groups

Significant differences in the morphometric variables were found between the fimbriae of the patients with UPSC and the patients with EEC (except for the Contrast and Correlation), as well as between each one of them and the group of healthy women.

#### Comparison of the morphometric textural features of the fimbriae, between healthy women and UPSC patients

**Fig 1:**
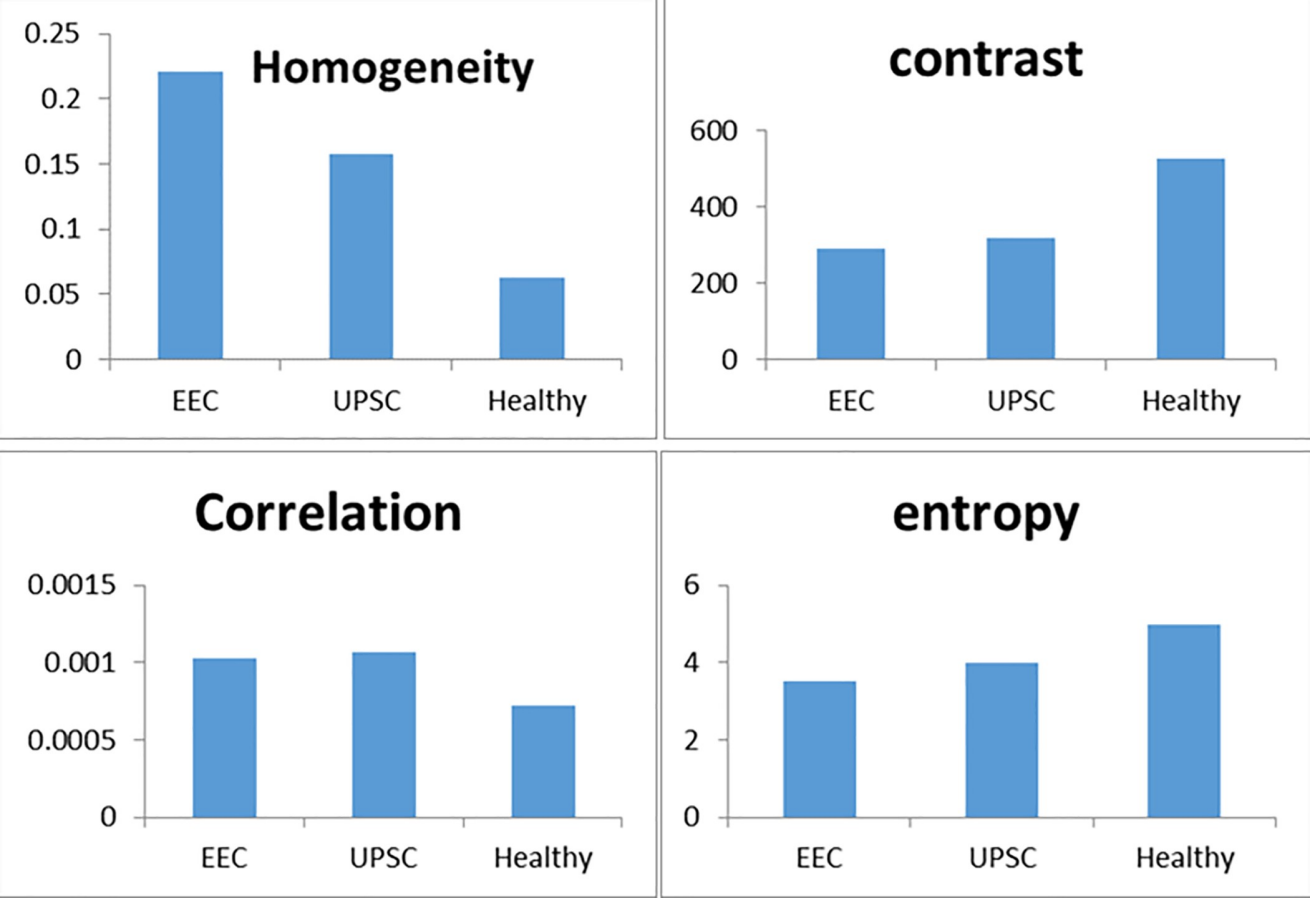
Fimbriae's structural changes in the three groups.

All four variables differed significantly between the fimbria of the healthy women and the patient with UPSC (homogeneity: 0.157± 0.041 vs. 0.063± 0.033, p<0.0001; contrast: 317.099 ± 110.294, vs. 525.1395± 133.139, p<0.0001 correlation: 0.001± 0.0002, vs. 0.0007± 0.0001, p<0.0001, entropy: 3.978± 0.346 vs. 4.965± 0.422, p<0.0001, for healthy and UPSC patients respectively). (Tables 1 and 2, Fig 1)

#### Comparison of the morphometric textural features of the fimbriae between healthy women and EEC patients

Tables 1 and 2 also show differences in the four textural morphometric variables: homogeneity, contrast, correlation, and entropy differed between the fimbriae of healthy women vs. patients with EEC (homogeneity: 0.220 ± 0.049 vs. 0.063± 0.033, p<0.0001; contrast: 292.106± 45.635, vs. 525.139± 133.139, p<0.0001; correlation: 0.001± 0.0001, vs. 0.0007± 0.0001, p<0.0001, entropy: 3.529± 0.334 vs. 4.965± 0.422, p<0.0001, for healthy and EEC patients respectively.

#### Comparison of the morphometric textural features of the fimbriae between patients with EEC and UPSC

Only two textural morphometric variables differed between the fimbriae from patients with EEC and patients with UPSC (Table 1: homogeneity: p = 0.001 and entropy: p = 0.002)

#### Univariate analysis

**Table 1:** The average variables for the patients in all three groups.

Group	Homogeneity	Contrast	Correlation	Entropy
**UPSC**	0.157± 0.041	317.099± 110.294	0.001±0.0002	3.978± 0.346
**EEC**	0.220±0.049	292.106±45.635	0.001±0.0001	3.529±0.334
**Healthy**	0.063±0.033	525.139±133.139	0.0007±0.001	4.965±0.422

**Table 2:** P Values.

P VALUES	Homogeneity	Contrast	Correlation	Entropy
**UPSC/healthy**	p<0.0001	p<0.0001	p<0.0001	p<0.0001
**EEC/health**	p<0.0001	p<0.0001	p<0.0001	p<0.0001
**EEC/UPSC**	0.001	0.551	0.516	0.002

#### Multivariate analysis

The only independent variable that was able to separate between all groups was the Homogeneity (Table 3).

**Table 3:** The predictive values of the Homogeneity variable.

Cross Validated		
	Sensitivity	Specificity
EEC vs Healthy	92.3%	95.80%
Serous vs Healthy	84.6%	91.70%
Serous vs EEC	76.9%	69.20%

### B. Analysis of the nuclear symmetry

Twenty-three relevant morphometric variables were included in our analysis.

**Fig 2:**
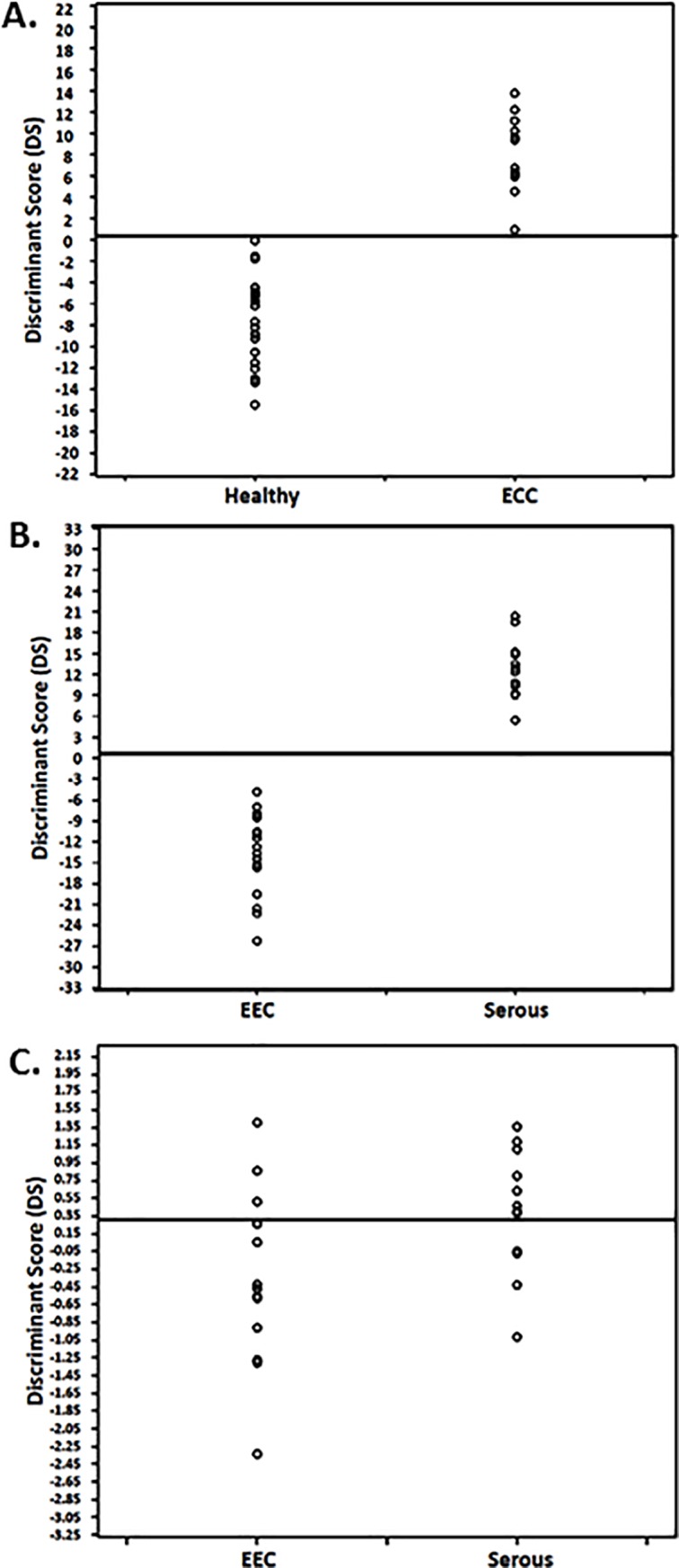
Discriminant scores and the cutoffs. **(A)** the discriminant scores and the cutoff for differentiating between healthy and EEC. **(B)** the discriminant scores and the cutoff for differentiating between healthy and UPSC.**(C)** the discriminant scores and the cutoff for differentiating between EEC and UPSC.

Three independent nuclear symmetry variables of the fimbria epithelial cells (minor axis, perimeter ratio and fractal dimension) have significantly separated between the healthy women and the EEC patients (Table 4). The regression coefficients resulting from the multivariate discriminate analysis and the values of the independent variables were used to compute a discriminant score (Equation #1) in order to differentiate between healthy and ECC patients with an optimal discriminant score cutoff equals to 0.4452. The patients with DS higher than 0.4452 were more likely to be associated with ECC (Sensitivity of 100%, as shown in Table 5) as compared to healthy women who showed DS values below this cutoff (specificity of 91.7%, as seen in Table 5). Fig 2A shows the DS values and the cutoff line for differentiating between the healthy and the EEC patients.

**Table 4:** The summary statistics of the independent predictors selected by the multivariate analysis.

Variable	Healthy (mean ± std. dev)	EEC (mean ± std. dev)	p-value
Axis (minor)	0.86191 ± 0.02831	0.91589 ± 0.01846	<0.000
Perimeter (ratio)	0.97577 ± 0.01286	0.98951 ± 0.00257	<0.000
Fractal Dimension	0.98607 ± 0.00871	0.9873 ± 0.00222	0.621*
Variable	Healthy (mean ± std. dev)	Serous (mean ± std. dev)	p-value
Area	0.86885 ± 0.04559	0.91542 ± 0.01431	<0.000
Diameter (max)	0.90599 ± 0.04258	0.94602 ± 0.01192	<0.000
Perimeter (ellipse)	0.92512 ± 0.02211	0.94169 ± 0.01483	.021
Perimeter (ratio)	0.97577 ± 0.01286	0.99139 ± 0.00175	<0.000
Variable	EEC (mean ± std. dev)	Serous (mean ± std. dev)	p-value
Perimeter (ratio)	0.98951 ± 0.00257	0.99139 ± 0.00175	0.039

* All these independent variables were statistically significant by univariate model.

**Table 5:** The predictive values of the discriminant score cutoffs.

Cross Validated		
	Sensitivity	Specificity
EEC vs Health	100%	91.70%
Serous vs Healthy	100%	95.80%
Serous vs EEC	61.50%	61.50%

In order to differentiate between the fallopian tube cells from healthy women versus UPSC patients, four nuclear symmetry variables were used (area, maximal diameter, perimeter-ellipse and perimeter-ratio), to compute a DS in order to divide between patients with serous carcinoma and healthy women (Equation #2 and Table 4) with a best DS cutoff equal to 0.3446. When the DS is greater than 0.3446, then we classified the patient as having serous carcinoma with a sensitivity of 100%. The healthy women displayed values mostly being lower than 0.3446 with a specificity of 95.8% (Table 5). Fig 2B shows the cutoff line for differentiating between serous papillary carcinoma vs healthy women.

When comparing the fallopian tube cells of ECC patients with UPSC patients, only one nuclear symmetry variable (perimeter-ratio) was found to differentiate between these two categories displaying a lower but still significant predictive power (see Equation #3 of the DS). Hence, patients with DS values greater than 0.3223 where more associated with serous carcinoma as opposed to DS values below 0.3223 that were more associated with EEC (both sensitivity and specificity of 61.5%, Table 5). Fig 2C demonstrates the cutoff for differentiating between the two cancer types.

**Fig 3:**
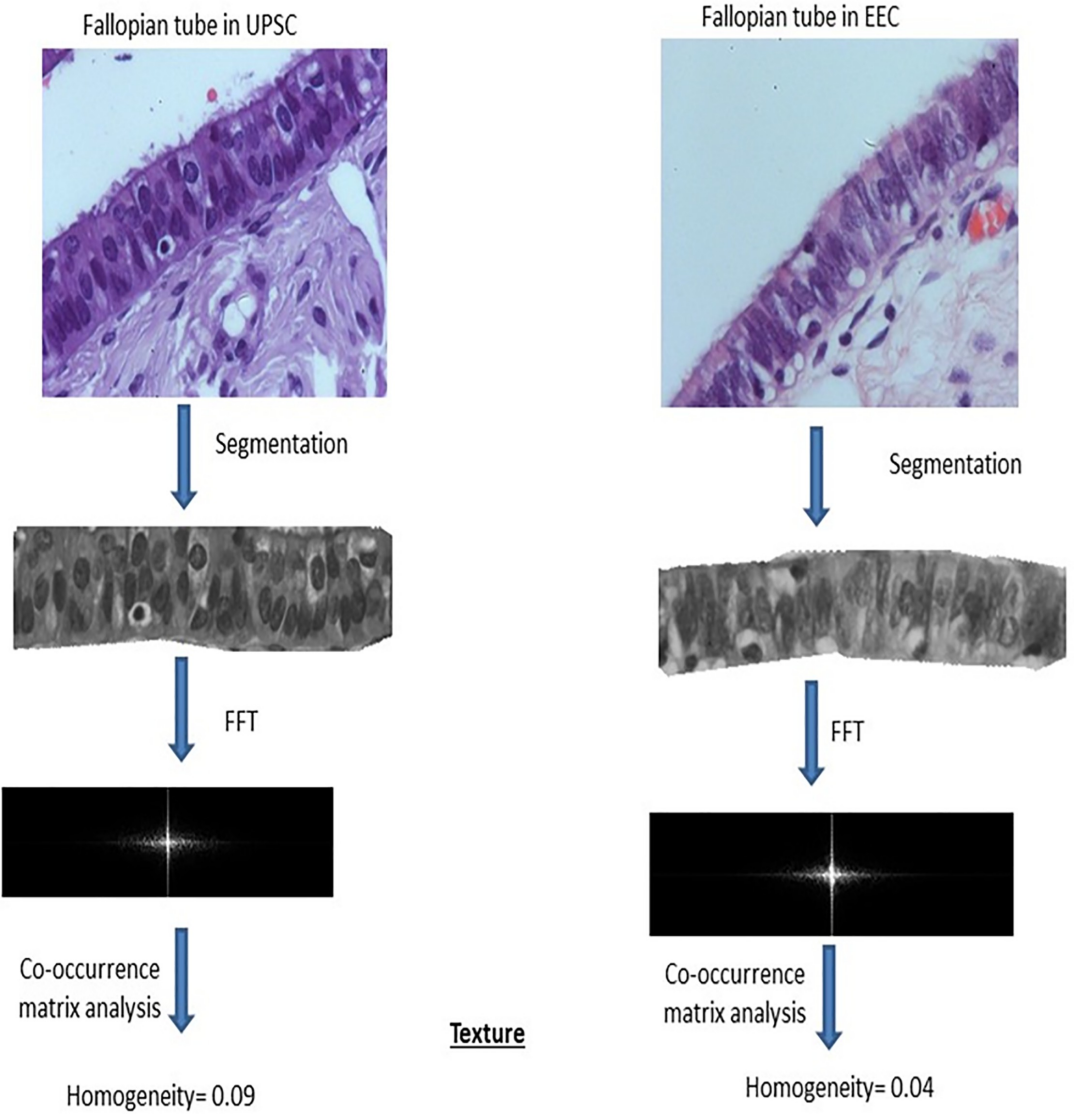
Difference in the architectural texture measured by homogeneity, in a USCP case versus an EEC case.

Fig 3 showing an example of the difference in the architectural texture measured by contrast, in a UPSC case versus an EEC case.

#### Univariate analysis

Multiple nuclear symmetry variables were statistically significant when compared between the groups (except between EEC and UPSC, that only 3 variables were significant: Perimeter (ratio), Density (min) and Density (std.dev.), therefore the summary statistics of the independent predictor selected by the multivariate analysis are presented in Table **4**.

Even though the Fractal Dimension has lost its significance in the last step of the multivariate model, we still chose to use it in the predictive model as recommended in specialized literature since the size effect of this variable is life and it significantly improves the predictive value of the equation.

Based on the independent predictors and their coefficients of regression, a discriminant score was computed (equations 1–3) as follows (presented in Table 5 and Fig 2):

Equation #1: The discriminant score for differentiating between healthy and EEC:

$$Ds=432.206+(axis_{minor}\times 227.854)+perimeter\_ratio\times 352.121)-(fracttal\;dimension\times 993.989)$$

Equation #2: The discriminant score for differentiating between healthy and UPSC:

$$Ds=-820.708+(area\times 259.131)+(diameter_{max}\times 190.784)-(perimeter_{ellipse}\times 367.444)+(perimeter_{ratio}\times 768.454)$$

Equation #3: The discriminant score (DS) for differentiating between EEC and UPSC:

$$Ds=(perimeter_{ratio}\times 388.602)-384.890$$

## Discussion

Understanding the pathogenesis of UPSC is important for early diagnosis, development of a dedicated treatment, and reduction of risks associated with the disease and its treatment [17].

There is a clinical and morphological similarity between ovarian serous papillary carcinoma and UPSC. The proximity of the endometrium to the fallopian tubes and the similar clinical behavior of serous endometrial cancer and ovarian cancer may suggest similar mechanism to carcinogenesis of both diseases. Jarboe et al reported 22 consecutive cases of uterine serous carcinoma in which serous tubal intraepithelial carcinoma (STIC) were found in 7 cases [18].Tolcher et al tried to determine if selected cases of uterine serous carcinoma arise from tubal rather than endometrial epithelium. They demonstrated endometrial intraepithelial carcinoma in 58% of patients with STIC and in 8% patients with USPC suggesting that some of the USPC may origin from the fallopian tubes [19]. Mu et al studied fallopian tubes of 30 patients with uterine serous cancer. They detected tubal epithelial lesions in 15 cases with positive expression of p53 in 87% out of endometrial malignant specimens’ tissues and 30% tubal tissues samples [20]).

Since the occurrence of some ovarian serous carcinomas was linked to a fallopian tube origin [11–13], we wondered if a similar possibility of an association between the tubal fimbria and UPSC could not be excluded.

In an attempt to test this hypothesis, we used novel methods of computerized morphometry to measure subtle morphological changes in the fallopian tube fimbria, including spatial orientation of the epithelial cells, using Fast Fourier Transformation followed by gray level co-occurrence matrix analysis and a second method of nuclear symmetry quantitation of the fimbria epithelial cells.

The FFT method has been already shown to be effective in distinguishing pre-malignant and malignant conditions in varying degrees in the gastrointestinal tract [14]. Using this morphometric analysis method, we could demonstrate the morphological changes in the fimbriae epithelium architecture (labeled as normal by the pathologist), obtained from women diagnosed with UPSC in comparison to those obtained from healthy women. These changes were statistically significant in each of the four variables examined (homogeneity, contrast, correlation, and entropy). The rationale for such analysis, based on these four variables, was to examine whether our findings have predictive value for the existence of UPSC in an otherwise normal fallopian tube.

Using different architectural and nuclear symmetry morphometric variables of the tubal fimbria epithelium, independently singled out by the multivariate analyses, accurate predictions could be performed, between healthy and UPCS as well as ECC as displayed in the results.

The finding of differences in the fimbria morphometric variables between healthy women and EEC patient is of an uncertain significance. On one hand, the uterine tumor may induce these subtle fallopian tube changes via hormonal factors and in the other hand, these fallopian tube changes may be very early precursors to predict UPSC. This question is especially important considering the fact that the fallopian tubes and the uterus epithelia display a common Mullerian origin [21].

In addition, a morphometric comparison of the fimbria epithelium was also performed between patients with UPSC and EEC. The variables that significantly differed between the tubes of these patients included homogeneity, entropy and many nuclear symmetry indices. Significant differences in many morphometric variables were also noticed between the fallopian tubes of these uterine cancers and tubes obtained from healthy women. Differences between UPSC and ECC were also reported by molecular studies. For example, a recent study mapped the genome methylation pattern of the two tumors in the uterus: UPSC and ECC, as well as of healthy endometrium. The researchers found significant differences that observed in the methylation patterns between the two tumor types, and the healthy endometrium [22]. This molecular data support the hypothesis that the pathogenesis of the two tumors is different based on evidence that DNA synthesis plays a role in cancer formation (Carcinogenesis). In fact, the study shows difference between the tumor groups and the normal (healthy) group and difference between the tumors themselves.

The fact that fallopian tube structural and nuclear symmetry changes differed among healthy women versus both uterine cancer patients, may suggest a common pathogenesis that may be linked to a fallopian tube origin of these types of cancer.

The pathogenesis of these cancers is not fully understood, and most studies are focusing on their treatment. The treatments offered today have indeed led to a reduction in mortality, but it still remained significantly higher than the other types of uterine cancer [23]. We assumed that a better understanding of the pathogenesis of these tumors may lead to an improvement in the prediction, prevention and treatment of these cancers. Additional studies comparing the fallopian tubes of women with endometrial hyperplasia with or without atypia to tubes from patients with endometrial carcinomas may help us to better differentiate between primary changes in the fallopian tubes and influence of the endometrial cell cancer on the fallopian tube epithelial morphology. However, such a study could only be meaningful if performed in patients with endometrioid cancer and not is serous papillary cancer.

## Supporting information

S1 File Raw data. (XLSX)
